# Whole-genome sequence dataset of *Pasteurella multocida* strain EH32 isolated from buffaloes in Vietnam

**DOI:** 10.1016/j.dib.2026.112699

**Published:** 2026-03-18

**Authors:** Thai Van Nguyen, T.T. Hang Trinh, Dinh Ng-Nguyen, Trong Van Nguyen, Hieu Quoc Nguyen, Van Duy Nguyen, Hung Vu-Khac

**Affiliations:** a Departerment of Veterinary Medicine, Faculty of Agriculture, Tay Nguyen University, Dak Lak, Vietnam; b Biotechnology department, Institute of Veterinary Research And Development of Central Vietnam, Khanh Hoa, Vietnam; c Institute of Biotechnology and Environment, Nha Trang University, Khanh Hoa, Vietnam; d School of Engineering, Newcastle University, Newcastle upon Tyne, UK

**Keywords:** Pasteurella multocida, Draft genome, Virulence genes, CAZymes, Vietnam

## Abstract

This Data in Brief article presents the draft genome of *Pasteurella multocida* strain EH32, isolated from a buffalo suspected of having septicemic pasteurellosis in Dak Lak Province, Vietnam. Short-read whole-genome sequencing (2 × 150 bp) was performed on the PacBio Onso platform, using an Illumina-compatible library. The final assembly comprised 2286,381 base pairs with a GC content of 40.2% across 25 contigs. Genome annotation predicted 2158 genes, including 2104 protein-coding sequences, four rRNAs, 49 tRNAs, and one tmRNA. Functional analysis assigned 2060 genes to COG categories and 1432 genes to KEGG pathways. Virulence-associated genes involved in adhesion, antiphagocytosis, endotoxin biosynthesis, immune evasion, iron uptake, secretion, and stress response were detected, while no antimicrobial resistance genes were found. A total of 105 carbohydrate-active enzymes were predicted, predominantly glycoside hydrolases and glycosyltransferases. Raw reads and assembly files have been deposited in Mendeley Data and GenBank. This dataset provides a foundational genomic resource to support comparative genomics and pathogenicity research on *P. multocida* in livestock.

Specifications Table

**Table:** 

Subject	Biology
Specific subject area	Bacterial genomics; infectious diseases in livestock; *Pasteurella multocida*
Type of data	Tables, Figure Raw, Analyzed, Filtered
Data collection	Genomic DNA of *P. multocida* strain EH32 was extracted from pure cultures using the QIAamp DNA Mini Kit. Sequencing libraries were prepared with the NEBNext dsDNA Fragmentase and NEBNext Ultra II DNA Library Prep Kit. Libraries were sequenced using a paired-end (2 × 150 bp) chemistry on the PacBio Onso platform. Genome annotation was performed with Prokka v1.14.6, and functional categories were assigned using the COG and KEGG databases; carbohydrate-active enzymes were identified with dbCAN3.
Data source location	University: Department of Veterinary Medicine, Faculty of Agriculture, Tay Nguyen University Ward/Province/Region: Buon Ma Thuot/Dak Lak/The Central Highlands Country: Vietnam
Data accessibility	1. Raw sequences Repository name: Mendeley Data Data identification number: DOI:10.17632/nyczcvbygc.1 Direct URL to data: https://data.mendeley.com/datasets/nyczcvbygc/1 2. Genome sequence Repository name : DDBJ/GenBank/EMBL Data identification number: JBRUXC000000000 Direct URL to data: https://www.ncbi.nlm.nih.gov/nuccore/JBRUXC000000000

## 1 Value of the Data

• This is the first publicly available genome dataset of a *P. multocida* serotype D strain isolated from buffalo in Vietnam.
• The dataset can be used for comparative genomics, phylogenetic analyses, and surveillance of *P. multocida* in livestock.
• Virulence and CAZyme profiles support research on pathogenicity mechanisms and vaccine development.
• The publicly available raw reads and annotated genome increase transparency and reproducibility in bacterial genomics research.

## 2 Background

Haemorrhagic septicemia caused by *P. multocida* in buffalo is a severe and rapidly progressing disease that results in high morbidity and mortality, leading to substantial economic losses in the livestock sector [1]. *P. multocida* is a Gram-negative, small, coccobacillary bacterium characterized by a prominent mucinous capsule [2]. In this dataset, *P. multocida* strain EH32 was isolated from a buffalo with suspected septicemic pasteurellosis in Dak Lak Province, Vietnam. Strain EH32 had been confirmed previously as capsular serotype D using a serotype d-specific PCR assay following [3]. Notably, infections in buffalo in Vietnam are predominantly associated with capsular serotype B [4,5], whereas genomic data for capsular serotype D isolates from Vietnamese buffalo remain limited. Therefore, we sequenced and annotated strain EH32 (capsular serotype D) to provide a publicly available draft genome resource for downstream comparative genomics and future epidemiological investigations. The dataset enables characterization of virulence-associated genes linked to adhesion, invasion, and infection. Additionally, key metabolic pathways were annotated using the KEGG and COG databases. This data supports comparative genomic studies and enhances understanding of the epidemiology and pathogenic potential of *P. multocida* in livestock. Therefore, the genome sequence and functional annotation of EH32 provide contextual genomic information for a capsular serotype D *P. multocida* isolate recovered from a buffalo in the Central Highlands of Vietnam.

## 3 Data Description

**Fig. 1:**
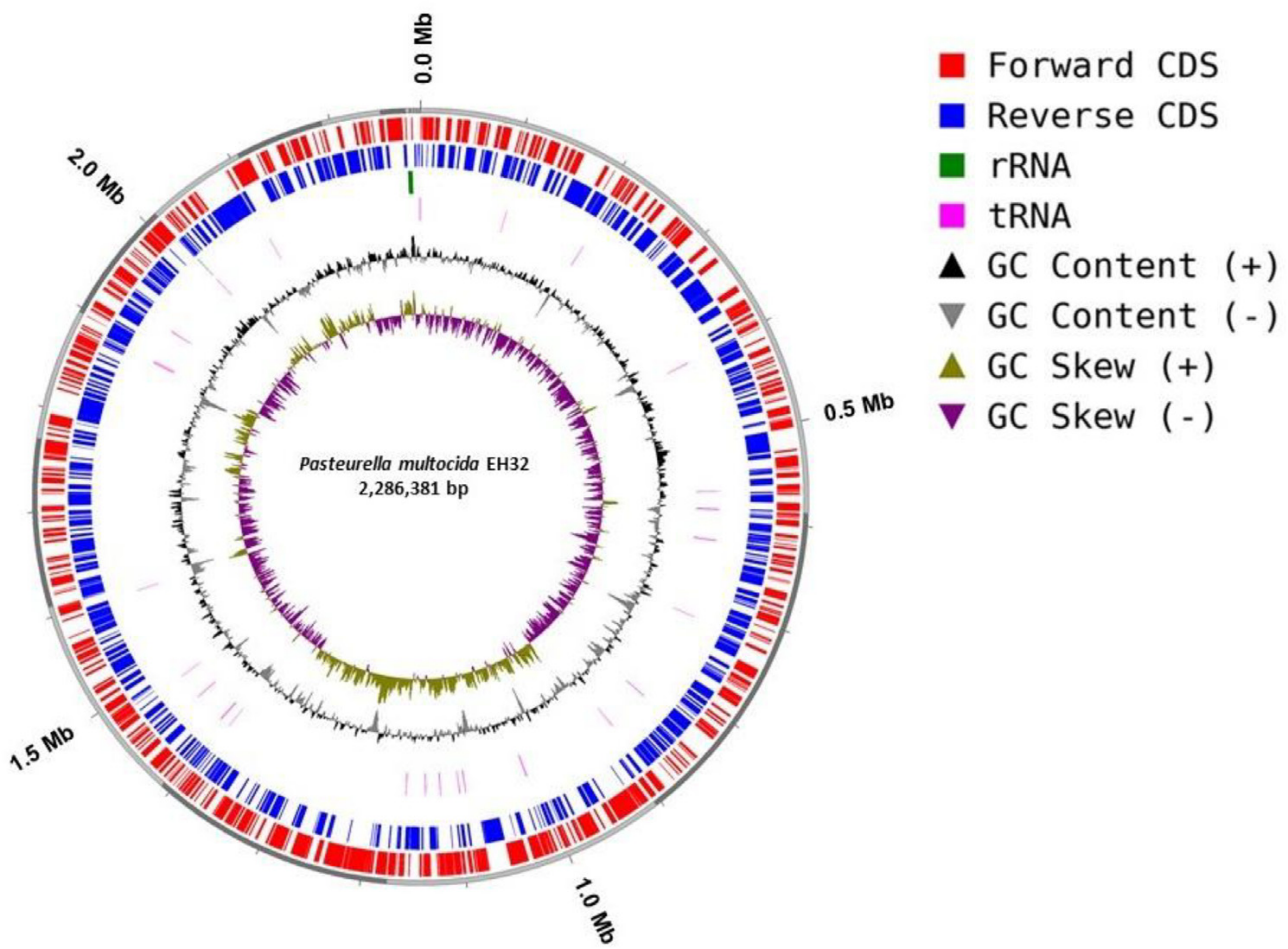
Circular representation of the draft genome of *P. multocida* strain EH32.

**Fig. 2:**
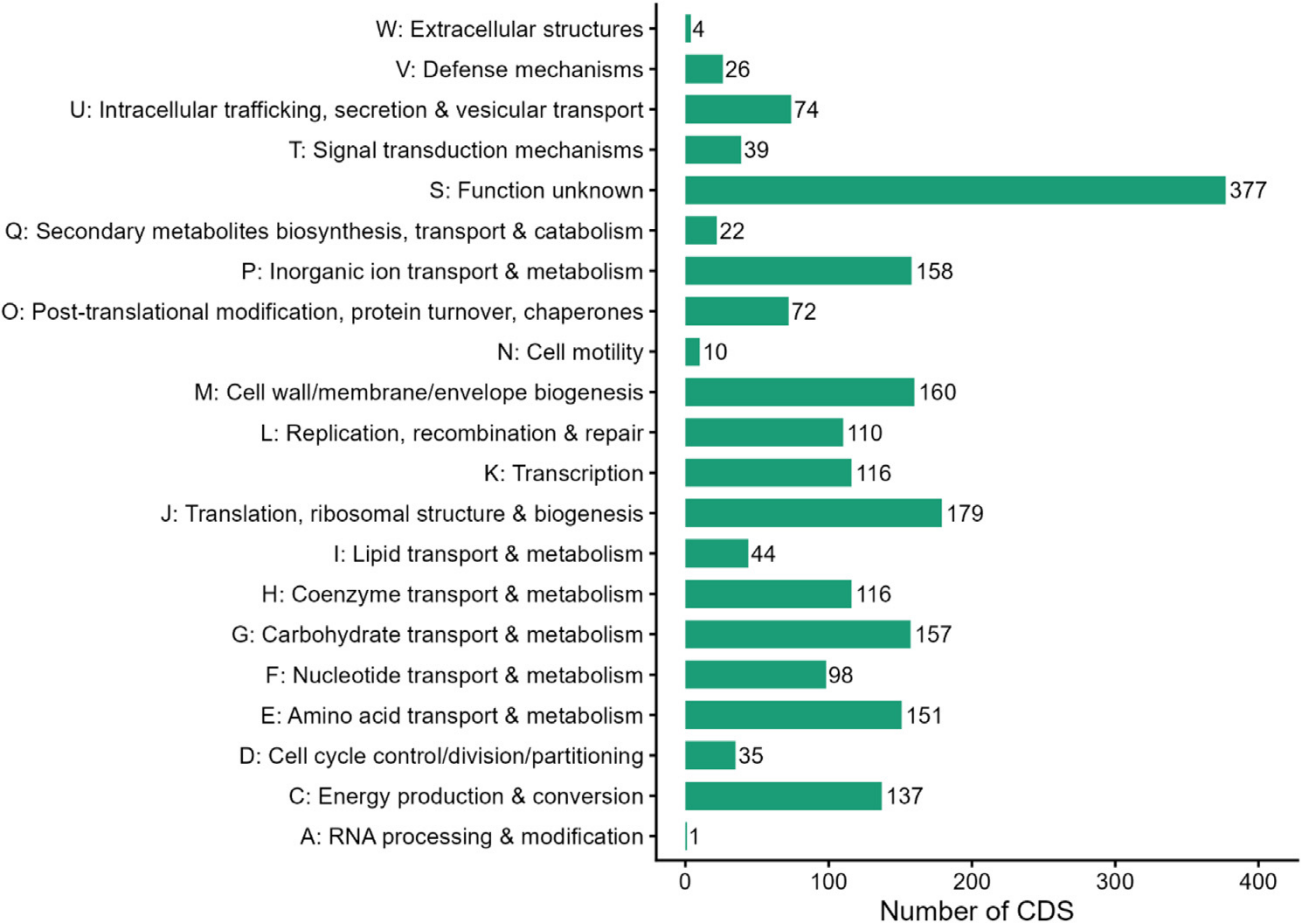
COG functional classification of coding sequences in *P. multocida* strain EH32.

**Fig. 3:**
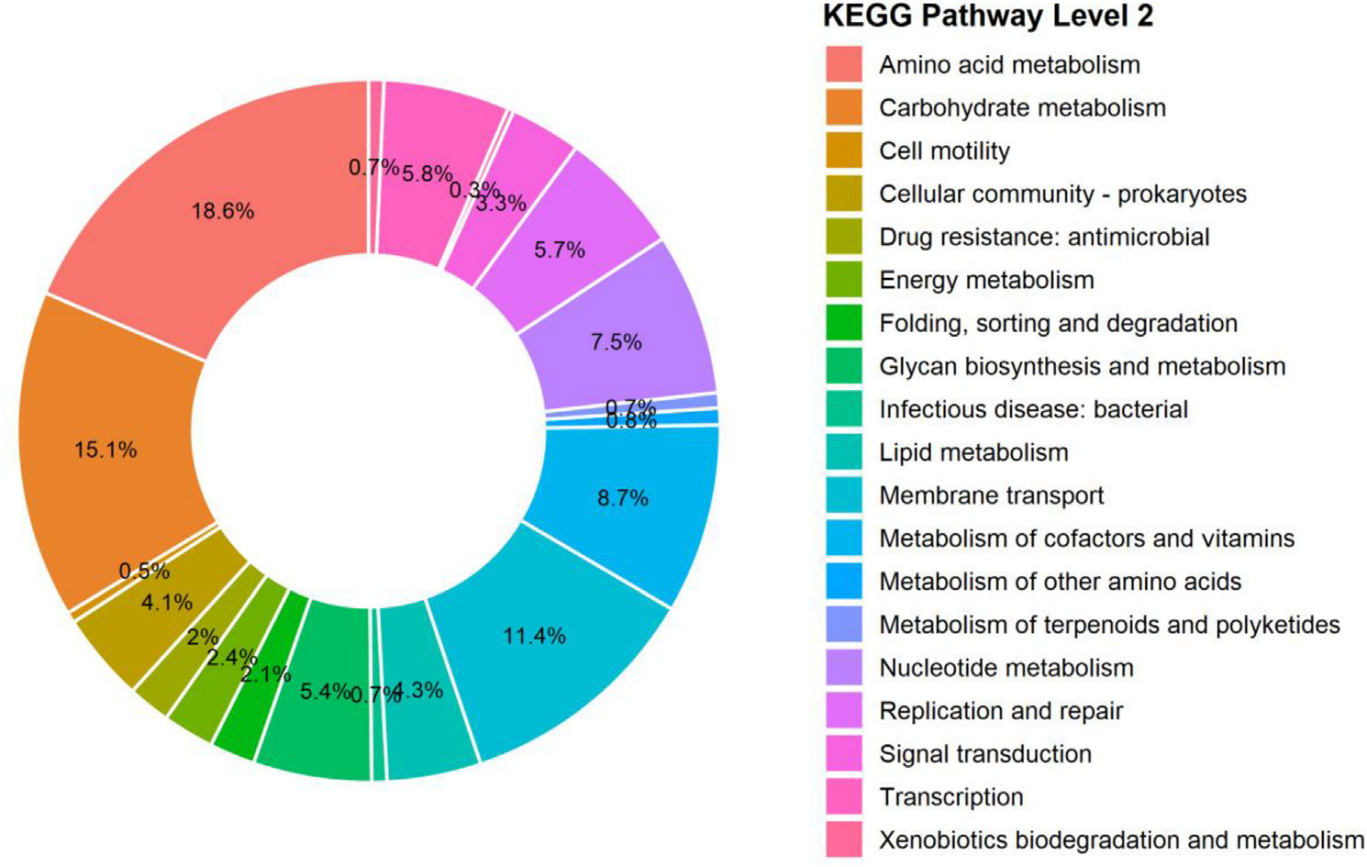
KEGG level 2 Pathway annotation of *P. multocida* strain EH32.

The dataset includes raw sequencing reads, the draft genome assembly, annotated genes, sets, and profiles of virulence factors and carbohydrate-active enzymes. Raw Illumina sequencing produced 4540,851 reads per direction (2 × 150), which, after trimming and quality control, resulted in 4373,562 clean paired-end reads (2 × 142 bp; 621,808,824 total bases) with a mean GC content of 40.3 % and high base quality (Q30 values of 99.6 % and 98.0 % for R1 and R2, respectively). De novo assembly initially produced 30 contigs; after filtering very short and low-quality contigs, 25 contigs were retained for downstream analyses, yielding a final draft genome of 2286,381 bp with a GC content of 40.2 %. CheckM estimated the genome completeness at 99.55 % with 0.02 % contamination. GTDB-Tk classified strain EH32 as *Pasteurella multocida*, with the closest reference genome GCF_900,187,275.1 (ANI 98.5 %, AF 0.9). This ANI value indicates high overall genomic similarity and is consistent with strong genomic conservation between EH32 and the closest reference genome, although the isolates originated from different hosts. However, ANI alone does not support conclusions regarding host adaptation, which would require broader comparative analyses across multiple-host-associated isolate. Genome annotation identified 2158 genes, including 2104 protein-coding sequences, four rRNA genes, 49 tRNA genes, and one tmRNA gene, and no pseudogenes were detected (Table 1 and Fig. 1). COG-based functional classification assigned 2060 genes (97.9 % of the predicted coding sequence), while KEGG pathway mapping assigned roles to 1432 genes (68.1 %) (Fig. 2 and Fig. 3). The raw sequencing data are publicly in Mendeley data (DOI:10.17632/nyczcvbygc.1), and the draft genome assembly has been deposited in DDBJ/GenBank/EMBL (accession: JBRUXC000000000).

**Table 1:** Genome features of *P. multocida* EH32.

Parameter	*P. multocida* EH32
Genome size (bp)	2286,381
*G* + *C* content ( %)	40.20
No. of contigs	25
The largest contig (bp)	587,197
Completeness ( %)	99.55
Contamination ( %)	0.02
N50	269,468
L50	3
Protein-coding sequences	2104
Hypothetical proteins	576
rRNA	4
tRNA	49
tmRNA	1

The outer ring indicates genome coordinates in megabases (Mb) with clearly labeled intervals. The outertracks show coding sequences (CDSs) on the forward and reverse strands, followed by rRNA and tRNA genes. The inner tracks display GC content and GC skew across the genome. The circular map was generated from the annotated draft assembly of EH32.

Bars indicate the number of predicted protein-coding genes assigned to each Cluster of Orthologous Groups (COG) functional category, summarizing the distribution of core metabolic, cellular, and information-processing functions in the EH32 genome.

Predicted protein-coding genes were mapped to Kyoto Encyclopedia of Genes and Genomes (KEGG) level 2 functional categories. The plot shows the number of genes assigned to each pathway category, highlighting major metabolic and cellular processes represented in the EH32 genome.

A total of 82 virulence-associated genes were detected in *P. multocida* strain EH32. These virulence-associated genes are reported as a presence/absence resource to support future comparative analyses with classical serotype B buffalo strains in larger genome collections. Fourteen genes associated with adherence were identified. Two capsule-associated genes and 32 genes involved in LPS/LOS biosynthesis were present. Twelve genes related to immune evasion and capsule formation, and 19 genes associated with iron uptake were recorded. Additionally, two oxidative-stress genes and one secretion-related gene were identified (Table 2). These gene categories are consistent with major pathogenicity-related functions described in *P. multocida*, including host adhesion, surface polysaccharide biosynthesis, immune evasion, iron acquisition, and stress adaptation.

**Table 2:** Summary of virulence-associated gene identified in *P. multocida* strain EH32.

Virulence category	No. of genes	Key factors	Functional notes
Adherence	14	*ompP5, flp1, flpD, rcpA, tadA-D, come/pillQ, pilA-C, lap, lptA*	Adhesion to host cells and epithelial colonization
Antiphagocyt- osis	2	*wza,wzb*	Capsule-associates immune evasion
Endotoxin	32	*gmhA/lpcA, htrB, kdkA, kdsA-B, kdtA, kpsF, lex2B, lgtA, lgtF, lic2A, lpxA-D, lpxH, lpxK, lsgA, lsgD, lsgE-F, msbA-B, neuA, opsX/rfaC, orfM, rfaD-F, rffG, waaQ, wecA*.	Lipooligosaccharide (LOS) and LPS core synthesis
Immune evasion	12	*galE, galU, manA-B, mrsA/glmM, pgi, ctrA-D, lipA-B*	Exopolysaccharide production and capsule formation
Iron uptake	19	*hitA-C, hemA-E, hemG-H, hemL-N, hemR, hemX-Y, hgpB-C, viuC*	Heme uptake and iron acquisition under host-limited condition
Secretion system	1	*ppkA*	Type VI secretion system (H-T6SS)
Stress resistance	2	*katA, sodC1*	Resistance to oxidative stress

A total of 105 carbohydrate-active enzymes (CAZymes) were identified in the genome of *P. multocida* EH32 (Table 3). These comprised 40 glycoside hydrolases (38.1 %), 57 glycosyltransferases (54.3 %), one polysaccharide lyase (0.9 %), four carbohydrate esterases (3.8 %), and three carbohydrate-binding modules (2.9 %). Thus, glycosyltransferases and glycoside hydrolases constitute the predominant CAZyme classes in EH32, whereas polysaccharide lyase, carbohydrate esterases, and carbohydrate-binding modules are only sparsely represented.

**Table 3:** CAZymes predicted in the *P. multocida* EH32 genome.

Class	Family (No.)
Glycoside hydrolases (GH)	GH0 (1), GH1 (3), GH3 (2), GH5 (2), GH6 (1), GH13 (12), GH18 (1), GH23 (3), GH24 (1), GH25 (1), GH28 (2), GH32 (1), GH33 (2), GH36 (1), GH77 (1), GH92 (1), GH102 (1), GH103 (2), GH131 (1), GH152 (1)
Glycosyl transferases (GT)	GT0 (1), GT1 (2), GT2 (13), GT4 (5), GT5 (1), GT8 (1), GT9 (5), GT25 (3), GT28 (1), GT30 (2), GT32 (1), GT33 (1), GT35 (1), GT45 (2), GT50 (1), GT51 (5), GT52 (1), GT58 (1), GT80 (1), GT100 (1), GT107 (1), GT119 (4), GT121 (2), GT129 (1)
Polysaccharide lyases (PL)	PL0 (1)
Carbohydrate esterases (CE)	CE4 (1), CE8 (1), CE9 (1), CE11 (1)
Carbohydrate-binding modules (CBM)	CBM50 (3)

No acquired antimicrobial resistance genes were detected using ABRicate/CARD when applying minimum identity and coverage thresholds of 80 %.

## 4 Experimental Design, Materials and Methods

The *P. multocida* strain EH32 was cultured on blood agar and incubated at 37 °C for 24 h. Genomic DNA was extracted using the QIAamp DNA Mini Kit (Qiagen, USA) according to the manufacturer’s instructions. Whole-genome sequencing libraries were prepared using the NEBNext dsDNA Fragmentase and NEBNext Ultra II DNA Library Prep Kit for Illumina (NEB, USA). Library concentration was measured using a Qubit fluorometer, and libraries ≥ 0.50 ng/µL were selected for sequencing. Sequencing was performed using 2 × 150 bp paired-end chemistry on the PacBio Onso platform.

Raw reads were filtered using fastp v0.23.1 to remove low-quality, ambiguous nucleotides, adapter sequences, and homopolymer regions [6]. Clean reads were assembled de novo using Unicycler v0.4.8 [7]. Assembly quality was assessed using Quast v5.2.0 [8] and read-to-contig mapping. Genome completeness and contamination were evaluated using CheckM v1.2.1 [9]. Genome annotation was performed using Prokka v1.14.6 [10]. Taxonomic classification was confirmed using GTDB-Tk v2.1.1 with the GTDB reference database [11,12]. Functional annotation (COG and KEGG assignments) was carried out on Galaxy using eggNOG-mapper v5.0.2 (Galaxy version 2.1.13+galaxy0). Virulence-associated genes were identified with VFanalyzer (VFDB). To screen for putative antimicrobial resistance genes, the assembled contigs were analyzed against the CARD database using ABRicate v1.0.1 (database version 27 March 2021), applying minimum coverage and identity thresholds of 80 % [13,14]. All plots were generated in R (version 4.4.3; R Core Team [15]) using the ggplot2 package [16].

## Limitations

Only a single isolate (EH32) was characterized, which may not capture the full genetic diversity of capsular serotype D circulating in buffalo in Vietnam.

The genome is currently available as a draft, multi-contig assembly rather than a fully circularized, closed chromosome.

## Ethics Statement

Ethical approval for this study and field sampling were obtained from the Animal Ethics Committee, Faculty of Animal Husbandry and Veterinary Medicine, Tay Nguyen University (Approval No. DTCB-2024). Biological sampling was conducted for research purposes from livestock with valid consent from all relevant stakeholders, including the animal owner and the local veterinary service, in accordance with applicable regulations and institutional guideline.

## CRediT Author Statement

**Thai Van Nguyen:** Conceptualization, Data curation, Formal analysis, Methodology, Software, Validation, Writing – Original Draft, Writing – review & editing. **T. T. Hang Trinh:** Data curation, Methodology, Validation, Writing – review & editing. **Dinh Ng-Nguyen:** Formal analysis, Methodology, Software, Validation, Writing – review & editing. **Trong Van Nguyen:** Methodology, Software, Validation**, Hieu Quoc Nguyen:** Methodology, Software, Validation**, Van Duy Nguyen:** Conceptualization, Software, Supervision, Writing – review & editing. **Hung Vu-Khac:** Conceptualization, Data curation, Methodology, Resources, Supervision, Visualization, Writing-review & editing.

## Data Availability

Mendeley DataWhole-genome sequencing of Pasteurella multocida strain EH32 isolate from buffalo in Vietnam (Original data).
